# Sleepiness and Health-Related Quality of Life Among Kidney Transplant Recipients in a Low-Middle Income Country: A Cross-Sectional Study

**DOI:** 10.3389/ti.2023.11547

**Published:** 2023-11-01

**Authors:** Mayssaa Hoteit, Ahmad Al-Masry, Martine Elbejjani, Mabel Aoun, Rana Abu-Dargham, Walid Medawar, Hilal Abou Zeinab, Laila Farhood, Sahar H. Koubar

**Affiliations:** ^1^ Division of Nephrology and Hypertension, Department of Internal Medicine, American University of Beirut, Beirut, Lebanon; ^2^ Division of Nephrology and Hypertension, Department of Internal Medicine, University of Iowa Hospitals and Clinics, Iowa City, IA, United States; ^3^ Clinical Research Institute, American University of Beirut, Beirut, Lebanon; ^4^ AUB Santé, Lorient, France; ^5^ Faculty of Medicine, Saint-Joseph University, Beirut, Lebanon; ^6^ Clemenceau Medical Center, Dubai, United Arab Emirates; ^7^ Division of Nephrology and Hypertension, Hammoud University Hospital, Saida, Lebanon; ^8^ School of Nursing, American University of Beirut, Beirut, Lebanon; ^9^ Psychiatry Department, Faculty of Medicine, American University of Beirut, Beirut, Lebanon; ^10^ Division of Nephrology and Hypertension, University of Minnesota, Minneapolis, MN, United States

**Keywords:** kidney transplant, sleepiness, quality of life, social support, low-middle income, BMI

## Abstract

This study aims to describe daytime sleepiness and health-related quality of life (HRQoL) among Lebanese kidney transplant (KT) recipients and to examine the medical, psychosocial and transplant factors related to them. It is a cross-sectional multi-center study involving KT recipients >18 years. Daytime sleepiness was assessed using ESS Questionnaire. HRQoL was measured using the SF-36 questionnaire. Social support was self-reported. A multivariable regression analysis evaluated factors associated with daytime sleepiness and HRQoL in our sample. 118 patients were recruited over a 2 years period. Excessive daytime sleepiness was prevalent in 12.7%. It was associated with Diabetes Mellitus (OR 3.97, 95% CI 0.94–16.81, *p* = 0.06) and obesity (OR 1.13, 95% CI 1.02, 1.27, *p* = 0.02). Social support and higher eGFR were associated with better scores on the MCS (β 24.13 *p* < 0.001 and β 0.26 *p* < 0.01) and the PCS (β 15.48 *p* < 0.01 and β 0.22 P 0.02). Conversely, depression and hospitalization were negatively associated with the MCS (β −27.44, *p* < 0.01 and β −9.87, *p* < 0.01) and the PCS (β −0.28.49, *p* < 0.01 and β −10.37, *p* < 0.01).

## Graphical Abstract

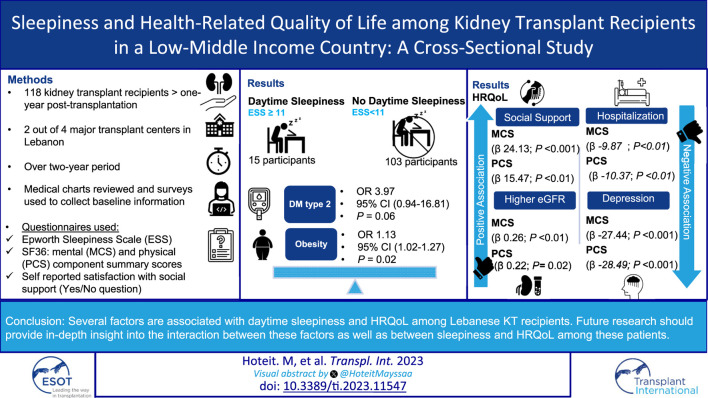

## Introduction

Kidney transplantation restores kidney function and alleviates many uremic symptoms and complications. Hence, sleep deficiency and poor sleep quality are less prevalent among Kidney transplant (KT) recipients than patients on maintenance dialysis. However, sleep deficiency and daytime sleepiness are still more common among KT recipients than the general population [1, 2]. In fact, poor sleep quality is widespread among KT recipients, with a frequency ranging from 30% to 62%, according to the Pittsburgh Sleep Quality Index (PSQI) [3]. Sleep disorders can either persist in chronic kidney disease (CKD) patients after transplantation, or develop as a *de novo* condition. Several biological and psychosocial factors may predispose KT recipients to increased prevalence of sleep deficiency and daytime sleepiness. In addition, immunosuppressive drugs can interfere with sleep and has been linked to non-adherence [4–6].

Regardless of the underlying sleep disorder, poor sleep quality and sleep deficiency culminate in daytime sleepiness. The latter can have a substantial impact on the health and wellbeing of patients. Nonetheless, it may also be associated with increased morbidity and mortality, as well as significant economic consequences such as increased healthcare utilization and lost productivity. Furthermore, poor sleep quality can weaken a KT recipient’s immune system. It has recently been established that even a minor disruption in sleep results in a reduction in natural immunological responses as well as a drop in the generation of T-cell cytokines [4, 7]. Hence, sleep deficiency is a critical matter for KT recipients. Proper screening and management may enhance patient survival, halt the progression of chronic kidney disease (CKD-T), and extend the much-needed graft survival, in addition to improving their Health-Related Quality of life (HRQoL) [6].

Another fundamental component of KT recipients’ wellbeing is HRQoL. For this particular population, HRQoL plays a crucial role in their overall satisfaction with life after transplantation and the impact of the transplant on their daily function and emotional state. HRQoL is rather a well-studied topic in KT recipients. Better HRQoL has been associated with better adherence to medications, enhanced social engagement and better long-term graft outcomes [8, 9].

Both sleep and HRQoL has biopsychosocial and cultural determinants. There is some evidence that older adults who possess robust social support networks might experience better sleep quality than those who do not [10]. Cultural factors such as eating habits, food content, race/ethnicity, gender roles, social class and cultural practices have been shown to affect sleep length [11]. Besides, cultural beliefs and values tend to shape our perception and experience of HRQoL. Factors such as social support, access to healthcare, spirituality and religion can influence our health behaviors and influence our perception of HRQoL. Thus, it is crucial to study sleep and HRQoL within the context of a specific culture/region.

Studies evaluating sleep disorders and HRQoL in KT recipients in the MENA region are almost non-existent. Lebanon belongs to the MENA region, which consists of geographically, and culturally close countries. Our study aims to describe daytime sleepiness and HRQoL among Lebanese KT recipients and to examine the factors linked to them, in order to better understand and serve this patient population.

## Materials and Methods

### Study Design, Settings and Participants

The study used a cross-sectional design, with participants recruited from the outpatient clinics of two major university hospitals in Lebanon over a 2 years period, from 1st June 2019 to 31st May 2021. Both hospitals are major transplant referral centers in the country, which has a total of four hospitals performing kidney transplants. Eligibility criteria were age >18 years old and kidney transplant performed >1 year at the time of recruitment.

Patients were first screened for eligibility by their primary nephrologist. Patients who agreed to participate, were contacted by the research assistant. If the participant had poor literacy, a trained research coordinator would go over the informed consent and questionnaires with them and a witness will co-sign the informed consent.

### Measurements

#### Outcomes of Interest

##### Daytime Sleepiness

This study aimed to determine the prevalence of daytime sleepiness and HRQoL among KT recipients who are ≥1 year post-transplant. The Epworth Sleepiness Scale (ESS) Questionnaire was used to assess the presence of daytime sleepiness. The ESS is a validated eight-item questionnaire to measure a subject’s expectation of dozing (falling into a light sleep) in eight situations. Dozing probability ratings range from 0 (no probability) to 3 (high probability) [12]. The score is divided into four categories: <6 is lower normal daytime sleepiness; 6–10 higher normal daytime sleepiness; excessive daytime sleepiness is above 10 and severe excessive daytime sleepiness is above 16. The participants were categorized into two groups: those scored < 11 as “Normal daytime sleepiness” group and those with a score ≥ 11 as “Excessive daytime sleepiness” group.

##### Health-Related Quality of Life

The Short-Form Health Survey (SF-36) was used to assess HRQoL perception. It is a widely used generic HRQoL measure with eight domains: physical functioning, role functioning-physical, role functioning-emotional, vitality, pain, general health, social functioning, and mental health [13]. The score for each domain ranges from 0 to 100. The higher the score, the more favorable the health outcome is. The SF-36 survey scores can be aggregated into two summaries: Mental component summary (which includes vitality, social functioning, role-emotional, and emotional wellbeing) and physical component summary (which include physical functioning, role limitations due to physical health problems, pain, general health).

Participants could choose between two languages: Arabic and English. Arabic is the official language of Lebanon and the MENA region. These instruments were chosen because they were the only ones validated in Arabic language [14–17] (Supplementary Material).

### Data Collection and Covariates

In addition to collecting demographic data, the survey collected data on several factors, which were grouped under several themes: psychosocial variables, medical variables including cardiovascular risk factors and co-morbidities, and kidney transplant related variables. Mental health scores for anxiety and depression were assessed using the Generalized Anxiety Disorder 7 Questionnaire (GAD7) and the Patient Health Questionnaire (PHQ9), respectively.

The GAD7 is a seven-item questionnaire used to assess the severity of anxiety. Participants were categorized into two groups based on their scores: “No/mild anxiety” for scores ≤ 9, and “Moderate/severe anxiety” for scores ≥ 10. The PHQ9 is a nine-item tool to screen and measure severity of depression. Participants were categorized based on their scores into two groups: “No/mild depression” for scores ≤ 9, and “Moderate/severe depression” for scores ≥ 10. Both anxiety and depression severity are based on the frequency of the occurrence of DSM-IV criteria in the previous 2 weeks.

We measured perceived social support. It was self-reported and evaluated using a single question “Are you satisfied with your social support?” with a yes/no answer. Demographic information (participant’s age, gender, educational background, marital and employment status and social habits including smoking and alcohol consumption) were self-reported. Information on the presence of comorbidities, body mass index (BMI), type of donor, time on dialysis, type of immunosuppression and laboratory data were retrieved from the patients’ hospital files.

### Ethical Considerations

The study was approved by the American University of Beirut’s institutional review board and carried out in accordance with the 1975 Helsinki Declaration. All participants signed an informed consent form before being included in the study.

### Statistical Analyses

Descriptive statistics were performed for all sample characteristics and outcome measures. For continuous variables, means and standard deviations (SDs) were reported and when non-normally distributed, medians and interquartile range (IQR) were reported. Categorical variables were described as numbers and percentages. When comparing participants with normal vs. excessive daytime sleepiness, comparisons of continuous variables were done using the independent t-test or the Mann-Whitney test and comparisons of categorical variables were based on Chi-Square test. We then performed age- and sex-adjusted logistic regression analysis to estimate association of each category of factors (psychosocial, medical and kidney transplant-related) with excessive daytime sleepiness defined by an ESS score ≥ 11. A multivariable logistic regression was then performed as a sensitivity analysis to assess the association of social support with excessive daytime sleepiness (taking into consideration two ESS thresholds: ≥10 and ≥11) by adjusting for medical indicators (BMI and diabetes, which were significantly associated with daytime sleepiness in age- and sex-adjusted models).

As for the HRQoL, internal consistency between the eight dimensions of the SF-36 questionnaire was evaluated by calculating Cronbach’s alpha. Age and sex adjusted linear regression analysis was done for sociodemographic and medical factors associated with the mental and physical component summaries of the SF-36 score (MCS and PCS). We had multiple variables that we were interested in and we wanted to run a consistent method for all, to facilitate comparisons as well as interpretations, and for most part, there was not large violations of using linear regression models. All analyses were conducted using Statistical Package for Social Sciences (SPSS), version 25.0.

## Results

### General Characteristics

A total of 124 adult kidney transplant recipients were approached to participate: 118 agreed to participate while six (5%) refused. The sociodemographic characteristics, comorbidities, kidney transplant related factors, mental health characteristics and SF-36 HRQoL scores of the participants are summarized in Table 1. Their mean age was 51 years and 66% were males. 91% of them were Lebanese while the remaining 9% were from neighboring arab countries. 72% were married and 57% were working. The mean age of the graft was 9.27 ± 6.5 years. 81% had hypertension, 13% had coronary artery disease (CAD) and 33% had diabetes mellitus (DM). Their mean BMI was 28.1 ± 5.39. 39% were hospitalized in the last year prior to recruitment. Their mean eGFR was 55.89 ± 21 ml/min/1.73 m [2]. Among our sample, 25% had moderate/severe anxiety while 14% had moderate/severe depression. The mean/median scores for the individual and summary components of the SF-36 HRQoL scores are listed in Table 1. 82% answered that they were satisfied with their social support.

**TABLE 1:** General characteristics of the total number of patients.

	Total *N* = 118
Socio-demographics	
Age, years, mean ± SD	51.01 ± 13.62
Sex, M/F, n (%)	78/40 (66/34)
Marital Status, n (%)	
Married	85 (72)
Other (divorced/single/widowed)	33 (28)
Number of children, mean ± SD	2.37 ± 2.22
Boy child, median (IQR)	1 [0, 2]
Girl Child, median (IQR)	1 [0, 2]
Number of grand-children, median (IQR)	0 [0, 0]
Occupational Status, n (%)	
Working	67 (56.8)
Other (retired/never worked)	51 (43.2)
Highest Educational level, n (%)	
University	48 (40.7)
Illiterate and school level	70 (59.3)
Satisfied with social support, n (%)	97 (82.2)
Medical factors/CV risk factors/Comorbidities	
Hypertension, n (%)	96 (81.4)
CAD, n (%)	15 (12.7)
Diabetes mellitus, n (%)	39 (33.1)
Dyslipidemia, n (%)	38 (32.2)
Cancer	5 (4.2)
Autoimmune disease, n (%)	3 (2.5)
Smoking status, n (%)	
Never	56 (47.5)
Ex-smoker	37 (31.4)
Current	25 (21.2)
Alcohol consumption, n (%)	
Never	99 (83.9)
Social	17 (14.4)
Drinker	1 (0.8)
Binge	1 (0.8)
Height, mean ± SD	166.69 ± 8.98
Weight, mean ± SD	79.25 ± 17.74
BMI, mean ± SD, Kg/m^2^	28.10 ± 5.39
Medical factors/Kidney transplant related	
Years of transplant, mean ± SD	9.27 ± 6.55
Type of transplant, n (%)	
Cadaveric	8 (6.8)
Living unrelated	44 (37.3)
Living related	66 (55.9)
Hospitalization last year, n (%)	46 (39.0)
Visit to psychiatrist, n (%)	4 (3.4)
Immunosuppression, n (%)	
Prednisone	112 (94.9)
Mycophenolate Mofetil	109 (92.4)
Cyclosporine	29 (24.6)
Azathioprine	2 (1.7)
Sirolimus	5 (4.2)
Tacrolimus	84 (71.2)
Everolimus	4 (3.4)
Regular medication intake, n (%)	118 (100)
Lowest serum creatinine, mean ± SD, mg/dL	0.96 ± 0.24
Delayed graft function, n (%)	4 (3.4)
Current serum creatinine, mean ± SD, mg/dL	1.50 ± 1.06
Current eGFR, mean ± SD, mL/min/1.73 m^2^	55.89 ± 20.59
Mental health and SF-36 quality of life scores	
Known mental health problem, n (%) (one anxiety, one bipolar, on medications, one improved)	2 (1.7)
Anxiety, n (%)	
None or mild	89 (75.4)
Moderate or severe	29 (24.6)
Depression, n (%)	
None or mild	102 (86.4)
Moderate or severe	16 (13.6)
Daytime sleepiness, n (%)	
Normal daytime sleepiness	103 (87.3)
Excessive daytime sleepiness	15 (12.7)
Physical functioning, mean ± SD	76.86 ± 26.87
Role limitations due to physical health, mean ± SD	77.12 ± 38.61
Median (IQR)	100 (50–100)
Role limitations due to emotional problems, mean ± SD	84.46 ± 33.67
Median (IQR)	100 (100–100)
Energy/fatigue, mean ± SD	62.12 ± 24.51
Emotional wellbeing, mean ± SD	69.92 ± 21.23
Social functioning, mean ± SD	84.43 ± 25.31
Median (IQR)	100 (74.25–100)
Pain, mean ± SD	81.05 ± 27.26
Median (IQR)	100 (66.88–100)
General Health, mean ± SD	59.92 ± 23.02
Health change, mean ± SD	64.62 ± 28.18
Mental Component Score (MCS), mean ± SD	75.23 ± 19.86
Physical Component Score (PCS), mean ± SD	73.74 ± 21.27

Note. Categorical variables are presented as numbers (n) and percentages (%). Continuous variables are presented as means and standard deviations (SD) if normally distributed and as medians and interquartile range (IQR) if skewed.

### Daytime Sleepiness

Fifteen patients (12.7%) had excessive daytime sleepiness while 103 (87.3%) had normal daytime sleepiness. There was no difference in age, gender, marital status, and educational status between the two groups. Those with excessive daytime sleepiness were more obese (mean BMI 32.4 ± 6 versus 27.48 ± 5.06, *p* < 0.01) and had more DM (67% versus 29%, *p* < 0.01). Among the different components of the SF-36 HRQoL scores, those with excessive daytime sleepiness scored significantly lower on physical functioning. The mean score for physical component score was 60.6 versus 75.6 (*p* = 0.01). Details about the differences between the two groups are summarized in Table 2. An Age and sex-adjusted logistic regression analysis (Table 3) followed by a multivariable logistic regression analysis showed that DM and BMI are associated with excessive sleepiness diabetes (OR 3.97, 95% CI 0.94, 16.81, *p* = 0.06) and obesity (OR 1.13, 95% CI 1.02, 1.27, *p* = 0.02, respectively) (Table 4).

**TABLE 2:** Comparison between those with normal daytime sleepiness versus those with excessive daytime sleepiness.

	Normal daytime sleepiness *n* = 103	Excessive daytime sleepiness *n* = 15	*p*-value
Socio-demographic factors			
Age, years, mean ± SD	50.91 ± 13.71	51.67 ± 13.38	0.84
Sex, M/F, n (%)	67/36 (65/35)	11/4 (73.3/26.7)	0.77
Marital status, n (%)			
Married	75 (72.8)	10 (66.7)	0.62
Other	28 (27.2)	5 (33.3)	
Number of children, mean ± SD	2.22 ± 2.04	3.4 ± 3.09	0.05
Number of grand-children, median (IQR)	0 [0, 0]	0 [0, 0]	0.50
Occupational status, n (%)			
Current or past working	95 (92.2)	14 (93.3)	0.78
Never worked	8 (7.8)	1 (6.7)	
Highest educational level, n (%)			
Illiterate/School level	58 (56.3)	12 (80)	0.09
University	45 (43.7)	3 (20)	
Satisfied with social support, n (%)	87 (84.5)	10 (66.7)	0.09
Medical factors/CV risk factors/Comorbidities			
Hypertension, n (%)	85 (82.5)	11 (73.3)	0.47
CAD, n (%)	12 (11.7)	3 (20)	0.40
Diabetes mellitus, n (%)	30 (29.1)	10 (66.7)	<0.01
Dyslipidemia, n (%)	34 (33)	4 (26.7)	0.77
Cancer, n (%)	5 (4.9)	0 (0)	0.99
Autoimmune disease, n (%)	3 (2.9)	0 (0)	0.99
Smoking status, n (%)			
Never	47 (45.6)	9 (60)	0.52
Ex-smoker	34 (33)	3 (20)	
Current	22 (21.4)	3 (20)	
Alcohol consumption, n (%)			
Never	87 (84.5)	12 (80)	0.71
Yes	16 (15.5)	3 (20)	
Height, mean ± SD, cm	166.47 ± 8.99	168.20 ± 9.03	0.49
Weight, mean ± SD, kg	76.96 ± 15.84	95.0 ± 22.29	<0.01
BMI, mean ± SD, kg/m^2^	27.48 ± 5.06	32.40 ± 5.87	<0.01
Kidney transplant related factors			
Years of transplant, mean ± SD	9.50 ± 6.57	7.66 ± 6.39	0.31
Type of transplant, n (%)			
Cadaveric and living unrelated	47 (45.6)	5 (33.3)	0.37
Living related	56 (54.4)	10 (66.7)	
Hospitalization last year, n (%)	41 (39.8)	5 (33.3)	0.63
Visit to psychiatrist, n (%)	4 (3.9)	0 (0)	0.99
Immunosuppression, n (%)			
Prednisone	97 (94.2)	15 (100)	0.99
Mycophenolate Mofetil	94 (91.3)	15 (100)	0.60
Cyclosporine	25 (24.3)	4 (26.7)	0.99
Tacrolimus	73 (70.9)	11 (73.3)	0.99
Lowest serum creatinine, mean ± SD, mg/dL	0.97 ± 0.25	0.97 ± 0.17	0.97
Delayed graft function, n (%)	4 (3.9)	0 (0)	0.99
Current serum creatinine, mean ± SD, mg/dL	1.46 ± 1.04	1.78 ± 1.22	0.35
Current eGFR, mean ± SD, mL/min/1.73 m^2^	56.75 ± 19.85	50 ± 25.11	0.33
Mental health and SF-36 quality of life scores			
Anxiety, n (%)			
None or mild	79 (76.7)	10 (66.7)	0.39
Moderate or severe	24 (23.3)	5 (33.3)	
Depression, n (%)			
None or mild	91 (88.3)	11 (73.3)	0.12
Moderate or severe	12 (11.7)	4 (26.7)	
Physical functioning, mean ± SD	79.71 ± 25.06	57.33 ± 31.50	<0.01
Role limitations due to physical health, mean ± SD	82.75 ± 34.39	45.83 ± 46.38	<0.01
Median (IQR)	100 (75–100)	25 (0–100)	
Role limitations due to emotional problems, mean ± SD	87.66 ± 30.22	66.68 ± 45.73	0.04
Median (IQR)	100 (100–100)	100 (0–100)	
Energy/fatigue, mean ± SD	62.33 ± 24.61	60.67 ± 24.56	0.81
Emotional wellbeing, mean ± SD	70.59 ± 20.85	65.27 ± 23.95	0.42
Social functioning, mean ± SD	86.37 ± 23.32	73.67 ± 33.14	0.15
Median (IQR)	100 (75–100)	100 (50–100)	
Pain, mean ± SD	82.51 ± 26.58	72.97 ± 30.30	0.26
Median (IQR)	100 (68–100)	80 (50–100)	
General Health, mean ± SD	59.42 ± 22.99	63.33 ± 23.73	0.55
Mental Component Score, mean ± SD	76.44 ± 18.91	66.92 ± 24.59	0.08
Physical Component Score, mean ± SD	75.64 ± 19.75	60.64 ± 26.94	<0.01

Note. Excessive daytime sleepiness is defined as ESS score ≥ 11. Continuous variables are compared using independent t-test and categorical variables are compared using Chi Square test.

**TABLE 3:** Age and sex-adjusted logistic regression analysis for psychosocial and medical factors associated with excessive daytime sleepiness.

	Age and sex adjusted		
Variables	OR	95% Confidence interval	*p*-value
Psychosocial factors			
Married	0.64	0.19, 2.16	0.47
Ref: other			
Number of children	1.25	0.99, 1.59	0.06
Education	3.05	0.81, 11.49	0.09
Ref: University level			
Working	1.03	0.30, 3.55	0.96
Ref: other			
Social support	0.353	0.09, 1.26	0.11
Depression	2.80	0.73, 10.76	0.13
Anxiety	1.62	0.50, 5.23	0.41
Comorbidities			
Hypertension	0.49	0.13, 1.84	0.29
CAD	1.76	0.40, 7.76	0.45
Diabetes mellitus	6.25	1.65, 23.61	<0.01
Dyslipidemia	0.66	0.18, 2.41	0.53
Smoking status: current	0.86	0.22, 3.38	0.83
Ref: other			
BMI	1.17	1.06, 1.30	<0.01
Kidney transplant-related factors			
Current serum creatinine	1.21	0.81, 1.79	0.35
Current eGFR	0.98	0.96, 1.01	0.26
Years of transplant	0.95	0.86, 1.05	0.33
Living related transplant	1.87	0.56, 6.26	0.31
Ref: Cadaveric and living unrelated			
Hospitalization last year	0.73	0.23, 2.32	0.59
CNI intake	0.61	0.12, 3.17	0.56

**TABLE 4:** Multivariable logistic regression analysis assessing factors associated with excessive daytime sleepiness.

	OR	95% Confidence interval	*p*-value
Social Support	0.27	0.07, 1.09	0.06
Age	0.97	0.92, 1.02	0.25
Sex	1.20	0.29, 4.92	0.79
Diabetes	3.97	0.94, 16.81	0.06
BMI	1.13	1.02, 1.27	0.02

### Health-Related Quality of Life

The internal validity of the different components of the SF-36 scores in our transplant sample was tested using Cronbach’s Alpha and it was very acceptable (Supplementary Table S1). All medical, psychosocial, and demographic factors were analyzed to assess their association with both the physical and mental component summaries of SF36 scores. Social support was associated with higher scores on both the mental and physical component summaries of the SF-36 (β 24.13, *p* < 0.01 and β 15.47, *p* < 0.01, respectively) as well as higher eGFR (β 0.26, *p* < 0.01 and β 0.22, *p* = 0.02, respectively). On the other hand, depression was associated with lower scores on both the MCS (β −27.44, *p* < 0.01) and the PCS (β −28.49, *<*0.01). The same applies to hospitalizations in the previous year (β −9.87, *p* < 0.01 and β −10.37, *p* < 0.01, respectively) (Table 5).

**TABLE 5:** Age- and sex-adjusted linear regression analysis for factors associated with the mental component summary and the physical component summary of the SF-36 health-related quality of life scores.

MCS				
	Standardized coefficient	Unstandardized coefficient B	95% CI for B	*p*-value
Married	−0.030	−1.327	−9.700, 7.046	0.75
Ref: other				
Working	0.054	2.173	−5.908, 10.255	0.59
Ref: other				
Education	0.068	2.75	−4.499, 9.996	0.45
Ref: university level				
Social support	0.467	24.130	15.520, 32.741	<0.001
Depression	−0.475	−27.440	−36.820, −18.060	<0.001
Smoking	−0.031	−1.501	−10.321, 7.318	0.73
Hypertension	−0.147	−7.450	−16.925, 2.024	0.12
Coronary artery disease	−0.093	−5.512	−16.645, 5.621	0.33
Diabetes	−0.027	−1.122	−9.300, 7.055	0.78
Dyslipidemia	0.024	1.001	−7.161, 9.162	0.81
BMI	−0.148	−0.545	−1.210, 0.120	0.11
Years of transplant	−0.037	−0.113	−0.687, 0.461	0.69
Living related transplant	−0.033	−1.303	−8.933, 6.328	0.73
Ref: other				
eGFR	0.267	0.258	0.086, 0.430	<0.01
Hospitalization last year	−0.243	−9.869	−16.989, −2.749	<0.01
**PCS**				
	**Standardized coefficient**	**Unstandardized coefficient**	**95% CI**	***p*-value**
Married	−0.013	−0.611	−9.692, 8.469	0.89
Ref: other				
Working	0.150	6.432	−2.258, 15.123	0.14
Ref: other				
Education	−0.057	−2.450	−10.313, 5.413	0.54
Ref: university level				
Social support	0.279	15.468	5.346, 25.590	<0.01
Depression	−0.461	−28.492	−38.784, −18.200	<0.001
Smoking	0.073	3.782	−5.758, 13.322	0.43
Hypertension	−0.101	−5.499	−15.829, 4.831	0.29
Coronary artery disease	−0.160	−10.185	−22.156, 1.787	0.09
Diabetes	−0.021	−0.959	−9.825, 7.908	0.83
Dyslipidemia	0.028	1.267	−7.581, 10.114	0.77
BMI	−0.125	−0.494	−1.217, 0.230	0.18
Years of transplant	−0.019	−0.062	−0.685, 0.560	0.84
Living related transplant	0.047	1.995	−6.273, 10.263	0.63
Ref: other				
eGFR	0.216	0.223	0.034, 0.412	0.02
Hospitalization last year	−0.239	−10.371	−18.105, −2.637	<0.01

### Social Support

84.5% of those with normal daytime sleepiness reported satisfaction with social support compared to 66.7% of those with excessive daytime sleepiness (*p* = 0.09). In a multivariate logistic regression, the association of social support with sleepiness varied according to the threshold of ESS used. When excessive daytime sleepiness was defined by a lower ESS threshold (score > 9), it was significantly associated with social support even after adjusting for depression (OR 0.17, 95% CI 0.04–0.75, *p* = 0.02) (Supplementary Table S2). With an ESS cutoff >10, satisfaction with social support was protective against excessive sleepiness but it did not reach statistical significance (OR 0.27, 95% CI 0.07–1.09, *p* = 0.06) (Table 4). This remained true when depression was added to the model (Supplementary Table S3). As for the HRQoL, social support remained significantly associated with the SF36 MCS score after adjusting-on top of age and sex-to depression (β 0.30, 95% CI 6.14–25.01, *p* < 0.01) (Supplementary Table S4).

## Discussion

Daytime sleepiness is rather prevalent among our KT recipients. Obesity and diabetes mellitus seem to contribute to it. Social support was positively associated with daytime sleepiness and its effect might be mediated by depression. Enhanced kidney function and perceived social support were linked with improved HRQoL ratings. Conversely, a history of hospitalization within the past year and the presence of depression exhibited connections with diminished HRQoL scores.

The prevalence of excessive daytime sleepiness in our transplant population was 12.7%. This is congruent with studies conducted in the general population, which revealed a prevalence ranging from 2.5% to 23% depending on the country of the study: 2.5% in Japan [18], 11% in Australia [19], 19% in Saudi Arabia [20], 21% in rural Canada [21], and 23% in Germany, [22]. There is not much published research on the prevalence and relevance of daytime sleepiness in KT recipients. The Epworth Sleepiness Scale data from three Swiss transplant hospitals found a 51% prevalence of daytime sleepiness [23]. This is significantly higher than our findings and could be explained by the lower ESS cutoff used (>6 vs. 11 in our study), as well as the older age of their study population (mean age 59 vs. 51). It is worth noting that the non-response rate in the Swiss study was 38%, with non-responders being much younger than responders. This could have contributed to the results being overestimated, as sleep difficulties are more common in older people. This is due to a combination of factors that come with aging rather than aging itself, such as physical and psychiatric illness, increasing medication use, changes in the circadian clock, and a higher prevalence of certain sleep disorders [24].

Our study showed that daytime sleepiness is positively associated with physical factors like obesity and DM. Weight gain is common during the first year after transplantation and thereafter [25]. Obesity (BMI ≥30 kg/m^2^) has been linked to fatigue and sleep disorders such as daytime sleepiness and obstructive sleep apnea (OSA), with obese participants being twice as likely as non-obese people to have excessive daytime sleepiness [26, 27]. In the transplant population, increased BMI is particularly important because it may have more serious implications, such as reduced graft survival and increased cardiovascular risk [28, 29]. The presence of OSA may have mediated the association we observed between high BMI and daytime sleepiness. Unfortunately, we were unable to determine whether patients were diagnosed with or treated for OSA.

Additionally, sleep disorders are highly prevalent among adults with DM [30]. In fact, sleep disorders may be a novel risk factor for the development of insulin resistance and DM. Getting sufficient sleep is essential for proper insulin secretion and glucose metabolism. This is particularly important in the transplant population who are inherently predisposed to steroid and immunosuppression induced DM. On the other hand, sleep disturbances can be induced by pain from common consequences of DM such as peripheral neuropathy or nocturia from inadequate glycemic control [30–32].

Although the results were not statistically significant, daytime sleepiness was positively associated with perceived social support. Mechanisms that may relate social support with better sleep quality include shielding against loneliness and social isolation, dampening stress levels, providing emotional support, embracing healthy sleep habits, and entraining circadian rhythms. On the other hand, sleepiness leads to less social interest, motivation and interactions. All these points indicate that the link between sleepiness and social functioning is possibly bidirectional with each entity influencing the other [33–35]. This association between daytime sleepiness and social support might have been mediated by depression. Indeed, studies have shown that depression is linked bi-directionally with sleep disorders [36], and since KT recipients are prone to depression [37], this could imply more sleep disorders in this group. As a result, clinicians are urged to look for the other comorbid disorder when sleepiness or depression are identified. Although we cannot draw solid conclusions from our cross-sectional design - due to its limited scope, which involved a single question to assess social support, a solitary subjective questionnaire to screen for daytime sleepiness, and reduced statistical significance-, it is important to underscore the potential role of social support in KT recipients in mitigating daytime sleepiness.

Along the same line, our study demonstrated a positive influence of social support on HRQoL scores within our cohort. This appears to be consistent across various countries. A French study evaluating HRQoL based on four fundamental dimensions—self-esteem, financial assistance, informational guidance, and emotional backing—revealed a significant association between deficient social support and lower HRQoL scores [38]. This correlation was observed in a Bahraini study as well, where married individuals exhibited notably higher HRQoL scores compared to their unmarried counterparts. This disparity was attributed to the additional social and financial support married participants received from their spouses and children, exerting a positive influence on their health and HRQoL [39]. Furthermore, a Chinese study identified social support, as gauged by the Social Support Rating Scale, as the primary determinant impacting adherence behavior and HRQoL [40].

Another positive link to higher HRQoL scores in our study was higher eGFR. This aligns with the outcomes observed by Legrand et al., wherein patients across all stages of CKD, including CKD-T, exhibited significantly lower physical HRQoL scores in comparison to the general population. Additionally, the adjusted mental component summary score was marginally lower among CKD-T patients compared to the general population, but statistically significant [41]. Furthermore, in a Japanese study involving KT recipients, scores of physical functioning, general health, and vitality closely correlated with serum creatinine levels. Individuals with a serum creatinine level exceeding 2 mg/dL displayed notably lower scores in contrast to those with levels below 1.5 mg/dL [42]. These findings illustrate the negative impact of graft dysfunction on HRQoL scores. On another hand, HRQoL scores are negatively impacted by recent hospitalization. A study by Gentile et al. found that hospitalization and recent critical illness were linked to worse HRQoL scores among KT recipients, similar to our findings [43]. Hospitalizations are linked to fatigue, deconditioning and anxiety; all these will affect HRQoL [44, 45].

Our study emphasized the well-known relation between depression and HRQoL in kidney transplant patients [46]. The experience of undergoing major surgery, managing complex medication regimens, coping with potential graft rejection, and adjusting to a new lifestyle can contribute to the development of depression in this population. Depression can affect their physical and emotional wellbeing as well as social and cognitive functioning. It can also interfere with adherence to medications, thereby affecting graft function and overall health [47]. Integrated care that includes psychological support, counseling, and, if necessary, pharmacological intervention, can play a pivotal role in managing depression and improving HRQoL in this patient population.

### Limitations and Strengths

The study’s main limitations were its cross-sectional design and small sample size. First, the cross-sectional design restricts our ability to establish whether daytime sleepiness observed post-kidney transplantation is a continuation of pre-transplant sleep problems or a new occurrence. Similarly, it hinders the ability to discern mediating or temporal links. Some features of social support, mental wellbeing, and quality of life may be overlapping and a cross sectional design may not capture the dynamic interaction between these issues. Second, our sample size was relatively small. It imposed limitations in detecting associations of smaller magnitude and we may have missed identifying other factors that contribute to the risk of sleepiness and deteriorating HRQoL. However, it is important to note that Lebanon is a small country with ∼1,000 kidney transplant recipients. Unfortunately, recruitment was hindered by the COVID-19 pandemic. Third, the study only included two transplant centers, however these are the major referral centers, serving a substantial portion of the country’s transplant recipients. Fourth, the absence of a healthy comparison group makes it challenging to determine whether the observed rates of sleepiness and HRQoL scores in the study sample differ significantly from those in the general population. Nonetheless, previous research has already demonstrated such differences. Fifth, the study relied on self-reported data for the assessment of daytime sleepiness and social support. Daytime sleepiness was evaluated through questionnaires rather than objective measures like polysomnographic sleep tests. Similarly, social support was perceived and evaluated based on a single question. While self-report screening questionnaires may not provide the same level of accuracy as objective diagnostic tests, they are efficient initial tools in any diagnostic process.

Despite these limitations, our study contributes valuable insights into the post-kidney transplant experiences of individuals in Lebanon, shedding light on important and often forgotten aspects of their post-transplant care such as sleep, social support, and HRQoL. The use of locally validated assessment tools among KT recipients may aid in the identification of those with excessive sleepiness or lower HRQoL scores, leading to the implementation of effective treatment strategies, to address these issues and improve their overall wellbeing.

## Conclusion and Future Directives

We have identified a variety of factors that are either positively or negatively associated with daytime sleepiness or HRQoL in kidney transplant recipients. Future research with larger and more diverse samples, longitudinal designs, and objective assessments could further elucidate the complex relationships among these variables. Exploring various dimensions of social support and their potential links to sleep disturbances warrants further investigation. Furthermore, a more in-depth understanding of the cultural differences contributing to sleepiness and HRQoL would enable the development of strategies to better address and manage these issues across diverse populations.

## Data Availability

The raw data supporting the conclusion of this article will be made available by the authors, without undue reservation.

## Abbreviations

BMI, Body Mass Index; CAD, Coronary Artery Disease; CKD, Chronic Kidney Disease; CKD-T, Chronic Kidney Disease in Kidney-Transplant; DM, Diabetes Mellitus; ESS, Epworth Sleepiness Scale; GAD-7, Generalized Anxiety Disorder; HRQoL, Health Related Quality of Life; IQR, Inter-Quartile Range; KT, Kidney Transplant; MENA, Middle East and North Africa; MCS, mental component summary score; OSA, Obstructive Sleep Apnea; PHQ9, Patient Health Questionnaire; PCS, physical component summary score; SD, standard deviations; SF 36, Short-Form Health Survey.
