# Supplementary material for: Sleepiness and Health-Related Quality of Life Among Kidney Transplant Recipients in a Low-Middle Income Country: A Cross-Sectional Study
Source: Transpl Int. 2023 Nov 1;36:11547. doi: 10.3389/ti.2023.11547 (PMC10647915; doi:10.3389/ti.2023.11547)

**Supplementary Tables:**

**Supplementary table 1(S1).** Reliability test of SF-36

|  | Cronbach's Alpha if item deleted |
| --- | --- |
| **Physical functioning** | 0.82 |
| **Role limitations due to physical health** | 0.80 |
| **Role limitations due to emotional problems** | 0.81 |
| **Energy/fatigue** | 0.81 |
| **Emotional well-being** | 0.82 |
| **Social functioning** | 0.80 |
| **Pain** | 0.81 |
| **General Health** | 0.82 |

**Supplementary table 2 (S2).** Comparison of different thresholds of the ESS scale

*If excessive daytime sleepiness score is considered above 9:*

|  | **OR** | **95% Confidence Interval** | ***p*-value** |
| --- | --- | --- | --- |
| **Model 1** | | | |
| **Social Support** | 0.20 | 0.06, 0.73 | 0.01 |
| **Model 2** | | | |
| **Social Support** | 0.17 | 0.04, 0.75 | 0.02 |
| **Depression** | 0.63 | 0.12, 3.70 | 0.63 |

Model 1 includes age, sex, diabetes, BMI; Model 2 includes Model 1 + depression

**Supplementary table 3(S3).** Multivariable logistic regression analysis assessing factors associated with excessive daytime sleepiness (adding depression)

|  | OR | 95% Confidence Interval | *p*-value |
| --- | --- | --- | --- |
| Social Support | 0.32 | 0.06, 1.69 | 0.18 |
| Depression | 1.43 | 0.24, 8.55 | 0.69 |
| Age | 0.97 | 0.92, 1.02 | 0.24 |
| Sex | 1.18 | 0.29, 4.85 | 0.82 |
| Diabetes | 4.11 | 0.96, 17.63 | 0.05 |
| BMI | 1,13 | 1.01, 1.27 | 0.03 |

**Supplementary table 4 (S4).**  Association of social support with the Mental Component summary score after adjustment to depression

| *Mental Component Score* | | | | |
| --- | --- | --- | --- | --- |
| Age- and sex-adjusted variable | Standardized  coefficient | Unstandardized coefficient | 95%CI | *p-*value |
| Social support | 0.301 | 15.578 | 6.142, 25.015 | 0.001 |
| Depression | -0.328 | -18.927 | -29.305, -8.549 | <0.001 |

Supplementary figure 1: Arabic Epworth Sleepiness Scale


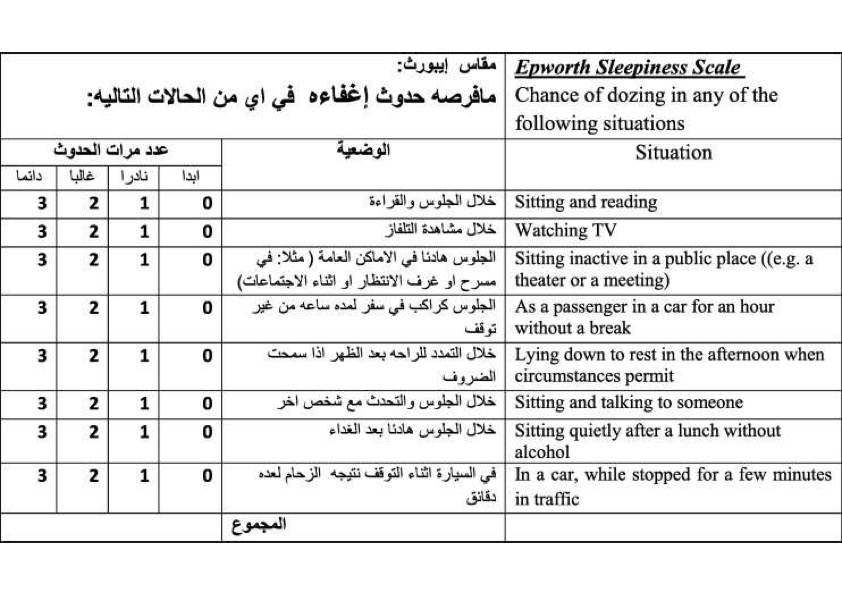


**Supplementary figure2: Arabic SF-36**


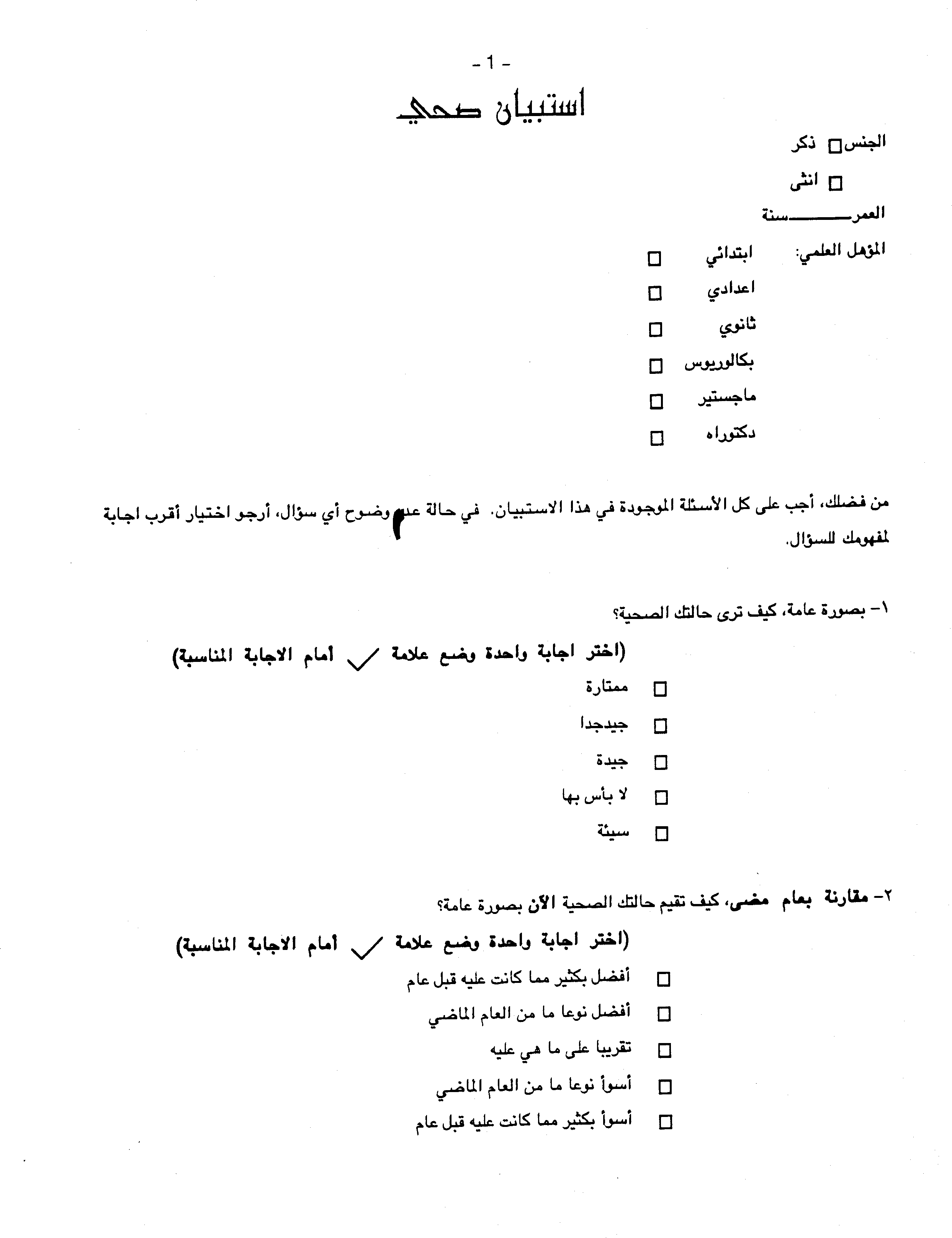


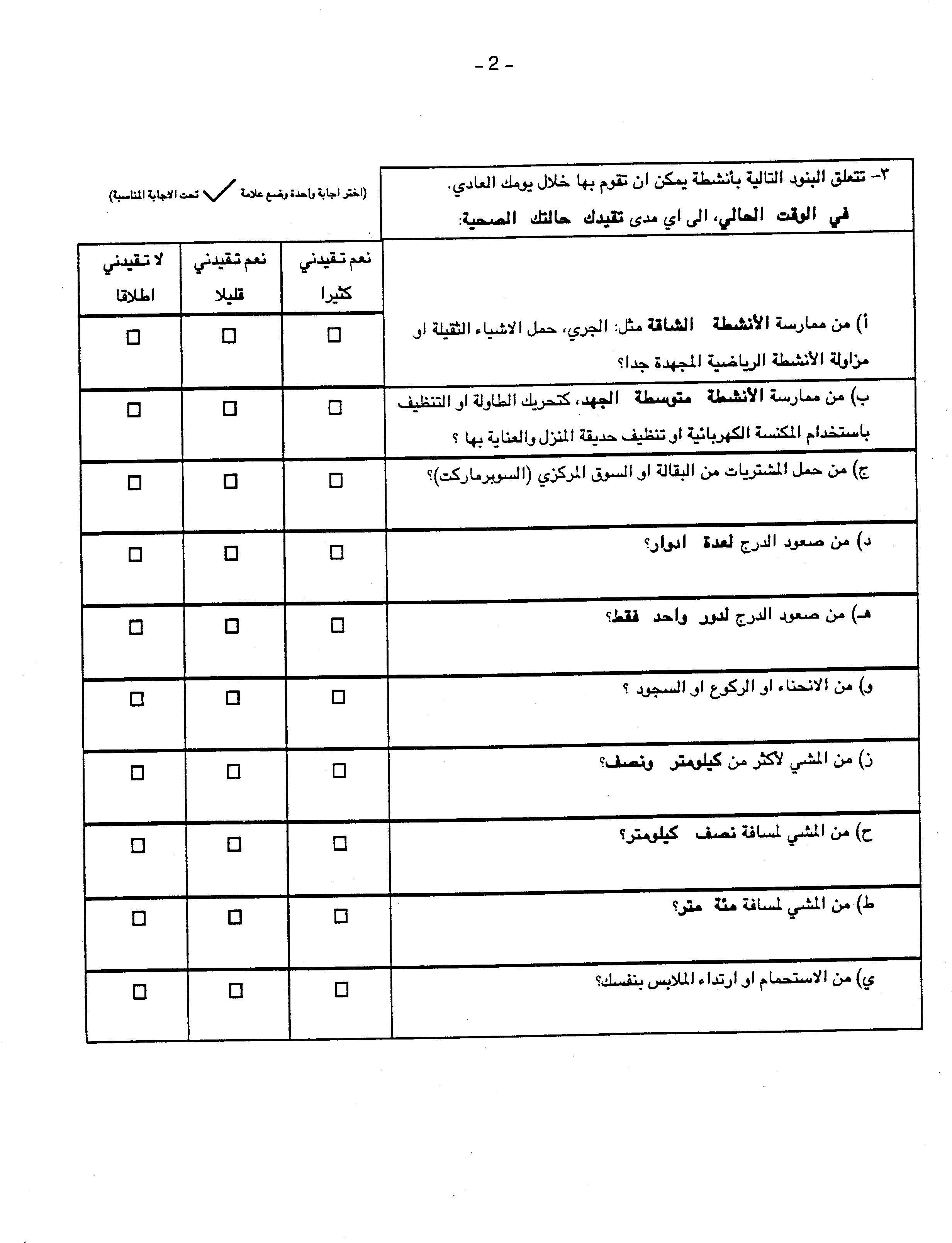


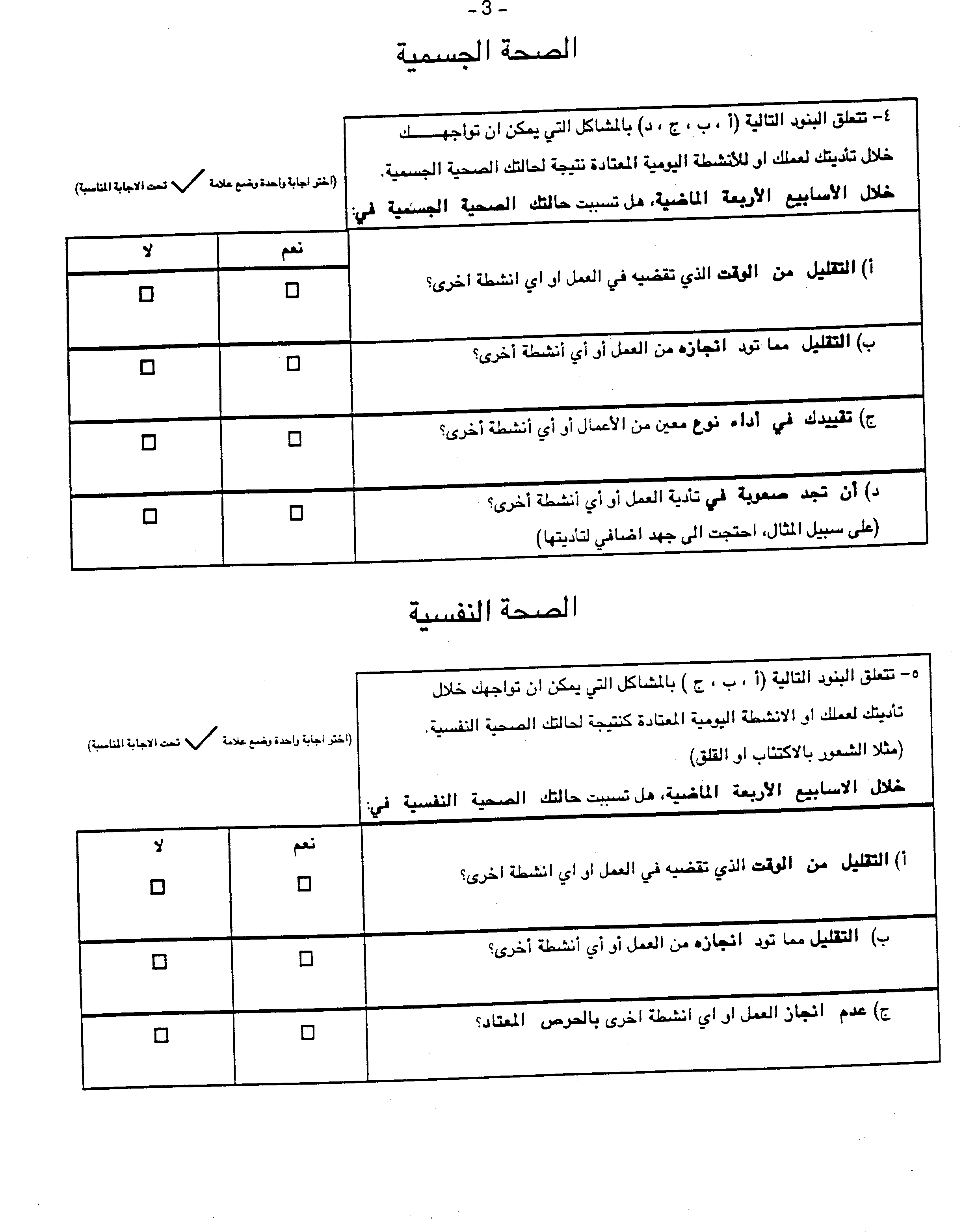


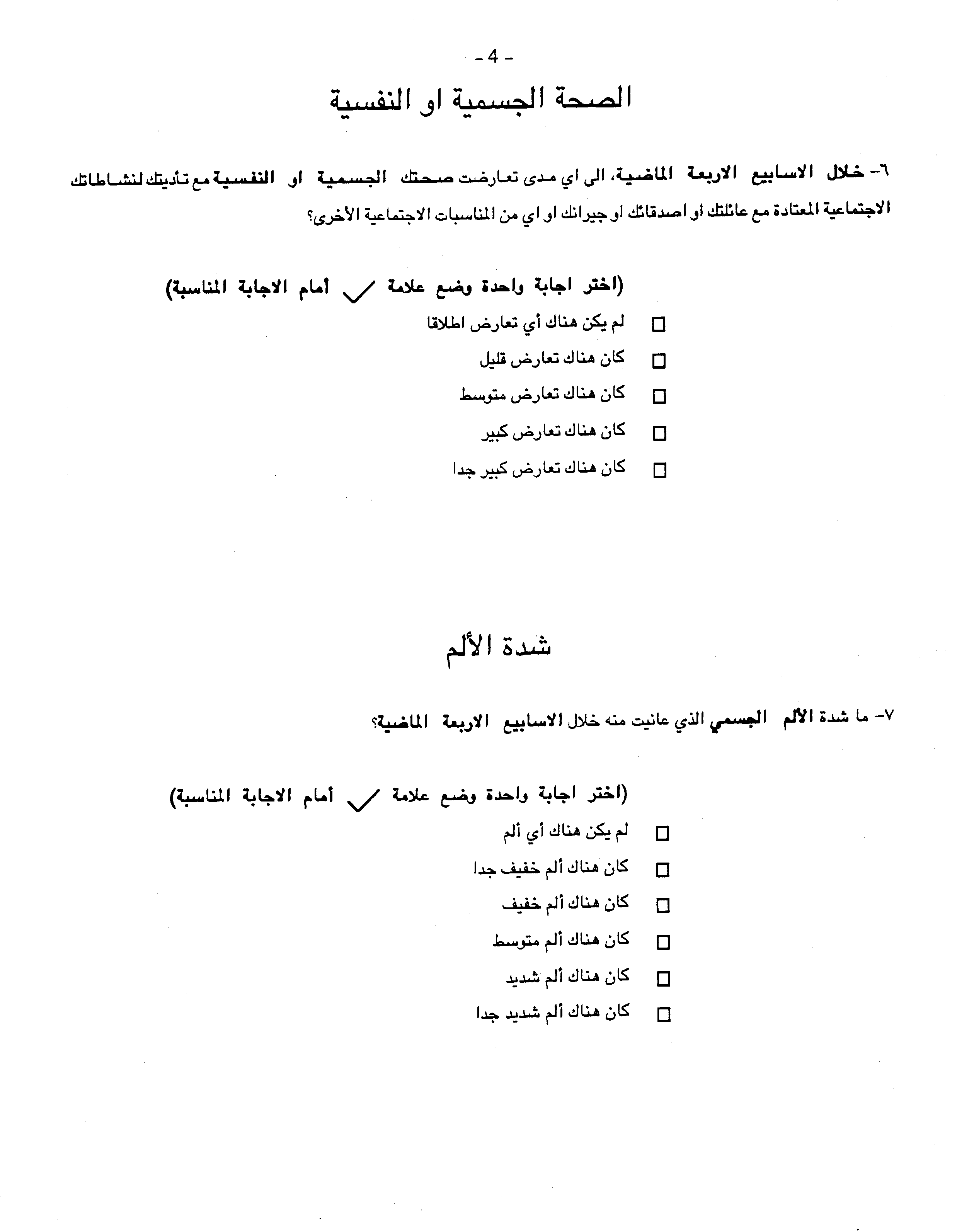


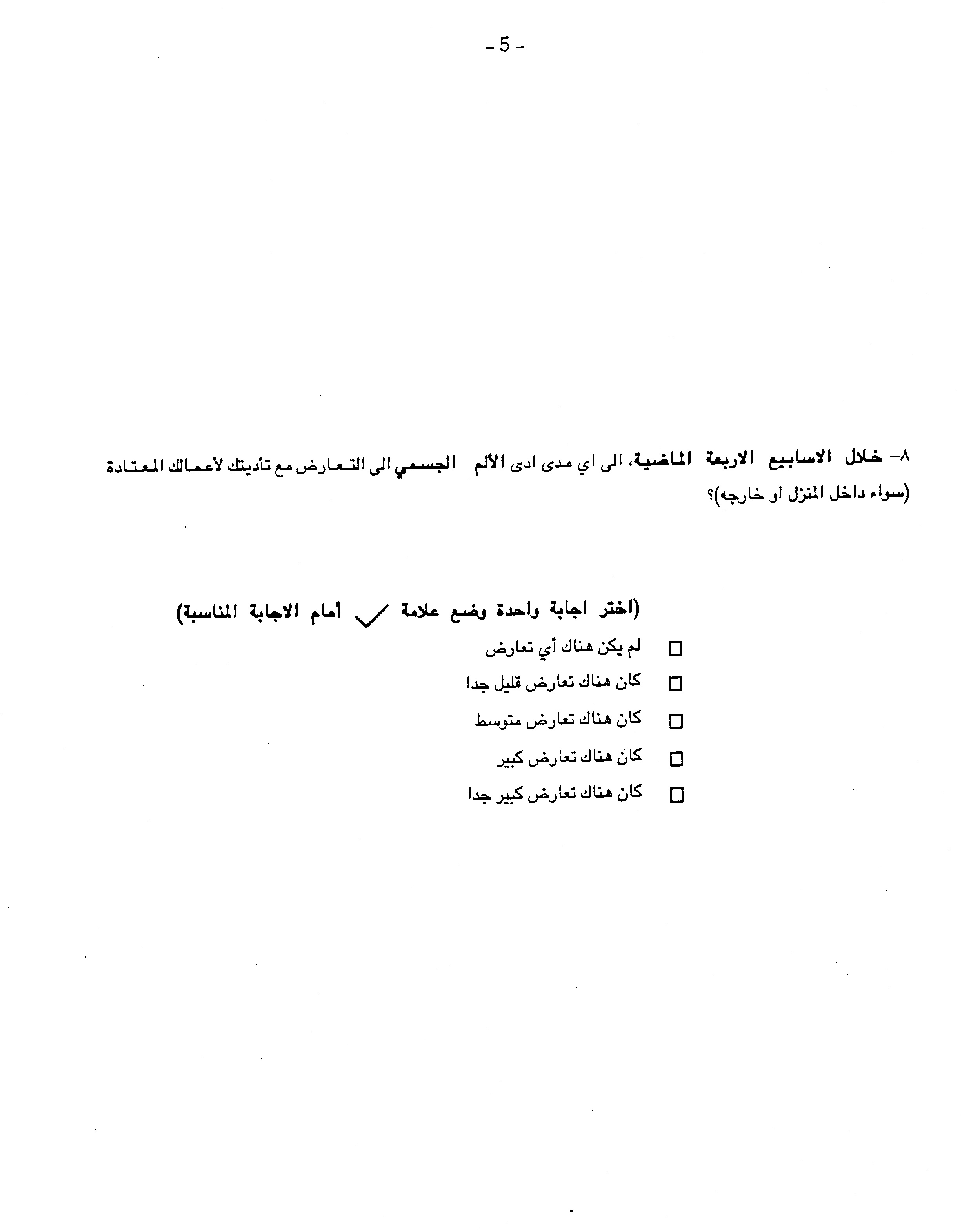


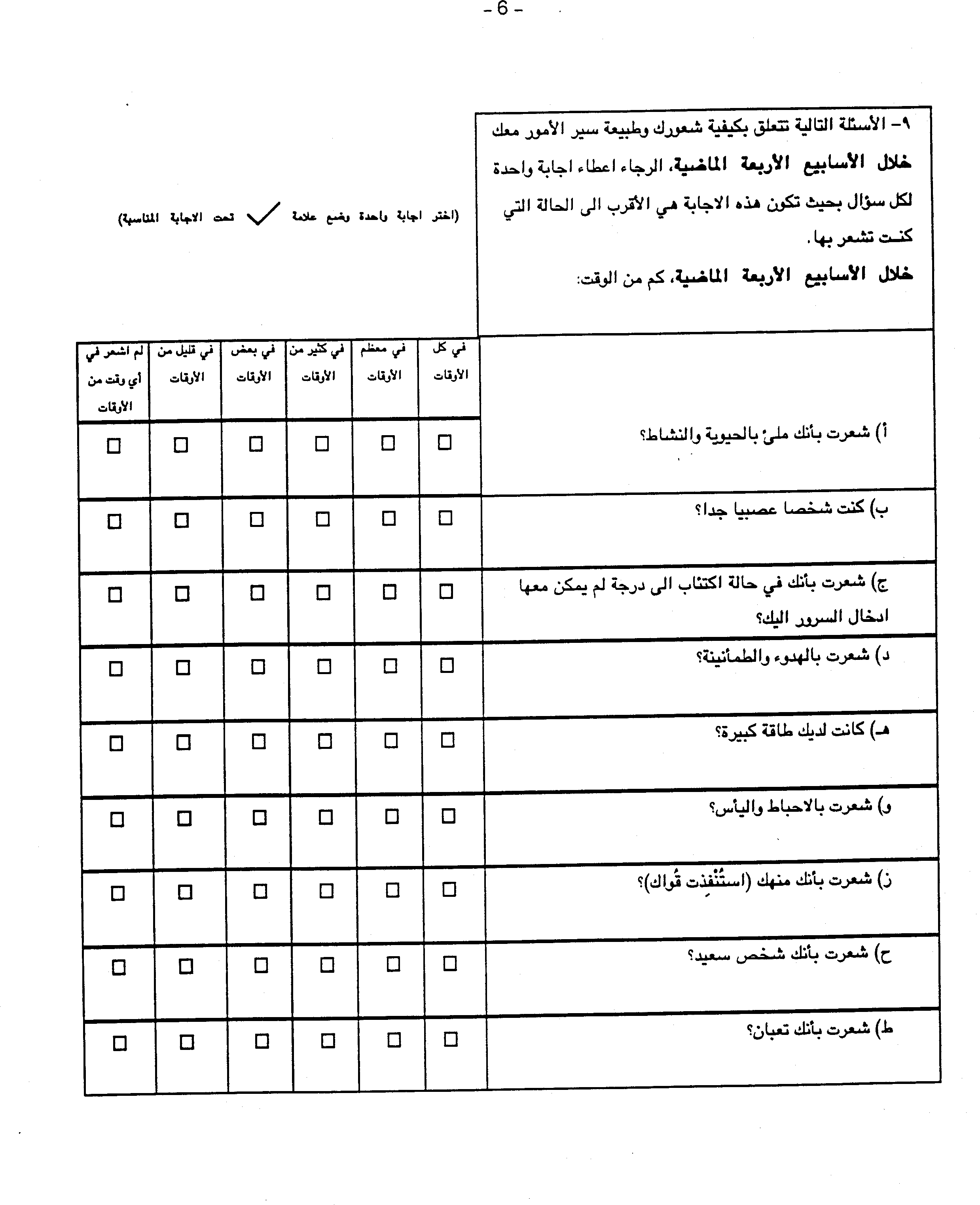


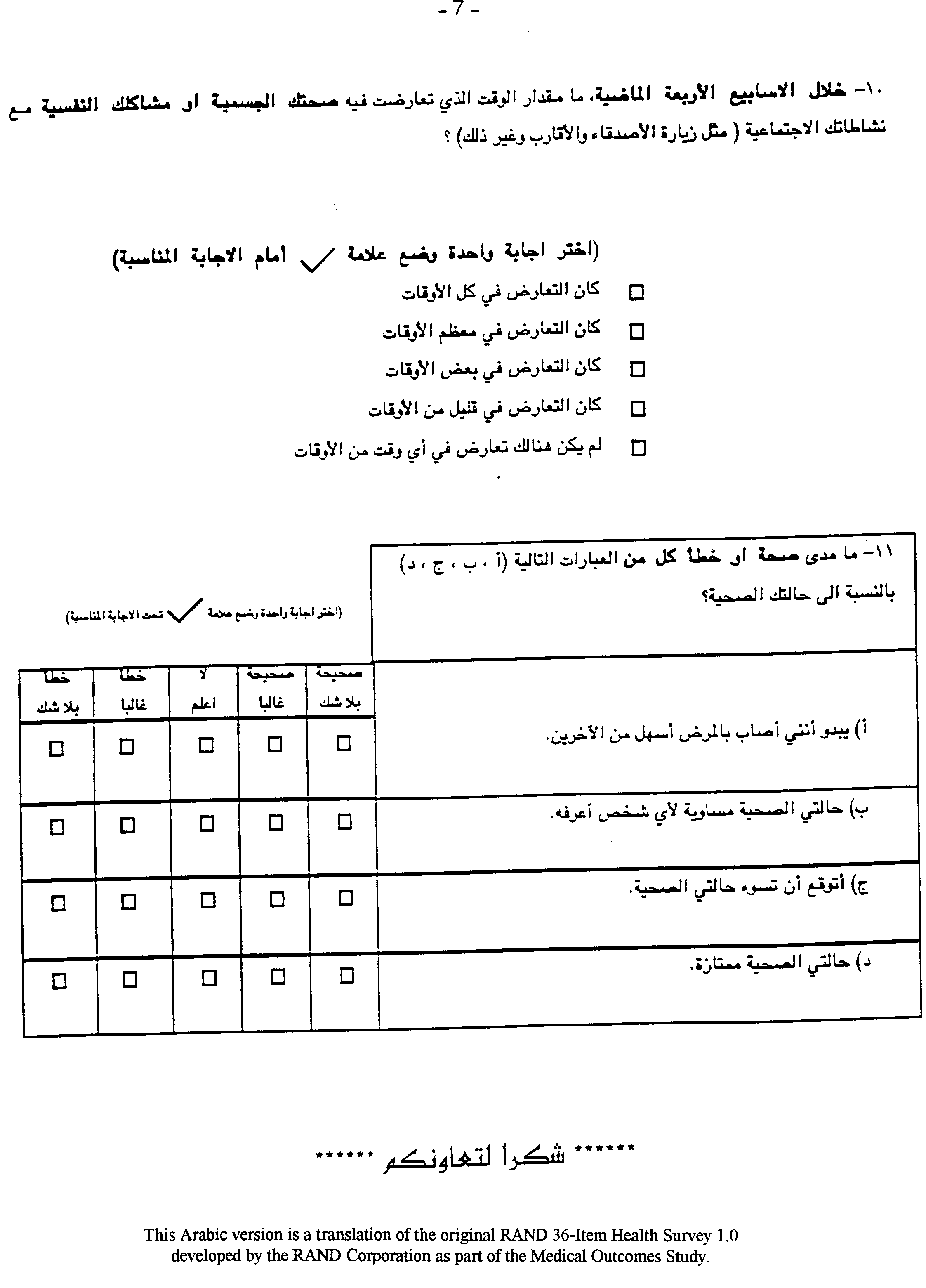

Supplement: Supplementary file 1 [file DataSheet1.docx]
